# Mendelian randomization studies on the causal relationship between insomnia and disease

**DOI:** 10.3389/fmed.2025.1709983

**Published:** 2026-01-21

**Authors:** Xuan Hou, Yingjun Wei, Yongfeng Wang, Lijun Wang

**Affiliations:** 1 Gansu Medical College, Pingliang, Gansu, China; 2 Gansu University of Chinese Medicine, Lanzhou, Gansu, China

**Keywords:** causal inference, insomnia, Mendelian randomization, review, risk factors

## Abstract

Insomnia, a prevalent sleep disorder, poses significant threats to both physical and mental health. Traditional studies suggest multiple factors are associated with insomnia, yet the causal direction often remains unclear and susceptible to confounding biases. Mendelian randomization, a cutting-edge method leveraging genetic instrumental variables for causal inference, effectively overcomes these limitations by providing high-quality evidence to clarify causal relationships between insomnia and various diseases. This review systematically integrates 105 recent Mendelian randomization studies on insomnia. Evidence indicates that insomnia exerts clear causal effects on multiple diseases, though the strength of these associations and the robustness of evidence vary by disease type. Insomnia is a robust risk factor for coronary heart disease, anxiety-depressive disorders, type 2 diabetes, and chronic pain. Causal relationships with osteoarthritis and lung cancer are also supported, though effect sizes are relatively small. Conversely, associations with Alzheimer’s disease and schizophrenia remain unconfirmed. The studies establish a dominant causal direction from “insomnia → disease,” effectively correcting potential reverse causality bias in observational research. These findings reposition insomnia from a common symptom to a key modifiable cause of a range of psychosomatic disorders. Causal inferences grounded in genetic evidence provide a robust scientific foundation for early identification of high-risk populations, precision prevention targeting insomnia, and cross-system comorbid management.

## 1 Introduction

Insomnia is a common clinical sleep disorder characterized by difficulty falling asleep, frequent awakenings during sleep, and difficulty returning to sleep after waking. Approximately 16.2% of adults worldwide experience insomnia to varying degrees, with nearly half of patients having a disease duration of ≥10 years (1). During the COVID-19 pandemic, global insomnia prevalence significantly increased (2), with 10% of recovered patients continuing to experience sleep disturbances (3). Chronic insomnia impairs neuroplasticity, manifesting as reduced synaptic density in the prefrontal cortex and decreased hippocampal neurogenesis (4, 5), while activating the stress-immune axis and elevating risks of depression and anxiety (6). Current first-line treatments include medication and cognitive behavioral therapy for insomnia (CBT-I). However, CBT-I coverage in primary care settings remains below 15% (7, 8), while benzodiazepine dependence rates reach 28–42% (9). Insomnia highly co-occurs with chronic conditions like depression, diabetes, and hypertension (10). However, randomized controlled trials (RCTs) face <5% applicability due to ethical constraints, while observational studies exhibit >30% causal reversal bias (11). Mendelian randomization (MR) leverages the random allocation property of genetic variants to simulate RCTs and reduce confounding (12, 13).

Mendelian randomization (MR) represents a novel research methodology for investigating causal relationships between exposure factors and outcomes. Grounded in Mendelian inheritance laws, MR employs genetic variants associated with risk factors as instrumental variables. These instruments must satisfy three core assumptions: (1) Strong correlation with the exposure factor (r^2^ > 0.01); (2) Independent of confounding factors; (3) Influencing the outcome solely through the exposure factor. It detects and explores associations between risk factors and diseases, mitigating the effects of confounding and reverse causality. By simulating randomized clinical trials through the principle of randomly assigned genetic variation, it provides insights into risk factors and drug targets to prioritize clinical trial investigations (14, 15).

The studies included in this review primarily employed inverse variance weighting (IVW) as the main method for estimating causal effects, as it provides the most precise estimates when all instrumental variables used are valid. To assess potential multi-effect bias, we focused on whether studies reported the following sensitivity analyses: MR-Egger regression (to detect and correct directional multi-effect bias), where an intercept *p* > 0.05 typically indicates negligible multi-effect bias; and weighted median method (which provides consistent estimates even if some instrumental variables are ineffective). We judged the reliability of evidence based on the following criteria: (1)Primary analysis method (IVW) *p* < 0.05; (2) Instrument strength assessed via *F* > 10 to mitigate weak instrument bias; (3) Sensitivity analysis results consistent with primary analysis direction and showing no significant heteroscedasticity (MR-Egger intercept *p* > 0.05). Results meeting all criteria were deemed robust causal evidence.

## 2 Search strategy

The literature search, screening, and data extraction process for this study strictly adhered to the guidelines outlined in the Preferred Reporting Items for Systematic Reviews and Meta-Analyses (PRISMA) statement. We systematically searched three Chinese databases—China National Knowledge Infrastructure (CNKI), VIP, and Wanfang Data—along with two English databases, PubMed and Springer Nature Link. The search period spanned from each database’s inception to December 31, 2024. The search strategy combined subject headings with free-text terms. The Chinese search query was “(Insomnia) AND (Mendelian Randomization Analysis OR MR),” The English search query, using PubMed as an example, was: ((“Insomnia”[Mesh]) OR (Insomnia[Title/Abstract])) AND ((“Mendelian Randomization”[Mesh]) OR (“Mendelian Randomization Analysis”[Mesh]) OR (MR[Title/Abstract]) OR (“Mendelian Randomization”[Title/Abstract])).

Inclusion and exclusion criteria were as follows: (1) Inclusion criteria: ① Study type was Mendelian randomization analysis; ② Insomnia was used as either the exposure or outcome variable; ③ Full-text Chinese or English articles were available. (2) Exclusion criteria: ① Duplicate publications; ② Inaccessible full-text articles; ③ Non-Chinese or non-English language; ④ Repeated analyses based on the same dataset; ⑤ Significant methodological flaws.

**Figure 1:**
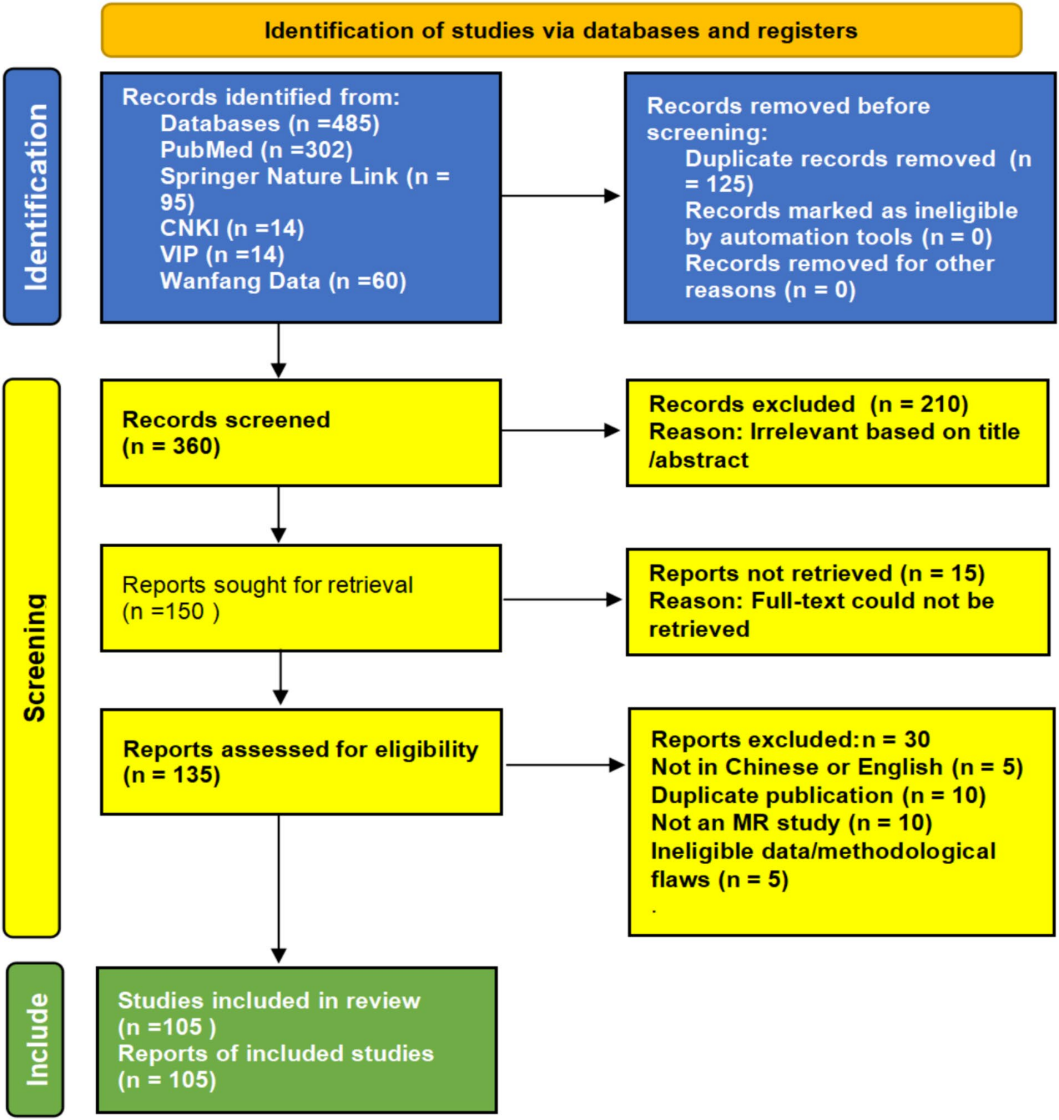
Retrieval strategy and research process diagram.

Literature screening and data extraction were performed independently by two researchers. First, retrieved articles were imported into reference management software for deduplication. Subsequently, researchers independently screened remaining articles based on titles/abstracts and full texts. Any discrepancies during screening were resolved through discussion, with arbitration by a third senior researcher when necessary. Ultimately, 105 articles were included in the analysis. The detailed process is illustrated in the PRISMA flow diagram (Figure 1).

To systematically assess the methodological quality of included studies, we employed the Newcastle-Ottawa Scale modified for Mendelian randomization studies (NOS-MR) for independent evaluation. This assessment primarily covered four core dimensions: strength of instrumental variables (e.g., reporting validity evidence such as F-statistic > 10), application of core MR methods (e.g., correct use of inverse variance weighting), sensitivity analysis and multi-effect testing (e.g., adequate use of MR-Egger and weighted median methods), and heterogeneity testing and robustness of results. Study quality was categorized as high (7–8 points), moderate (5–6 points), or low (≤4 points) based on total scores, with specific criteria detailed in Table 1 footnotes. The assessment revealed that the overall quality of the included studies was above average, with most scores ranging between 5 and 8 points (Table 1). This indicates relatively adequate methodological reporting and robust core findings. Data extraction was conducted concurrently, capturing information including study characteristics, definitions of exposure and outcomes, details of instrumental variables, types of MR analysis, and primary results. This information was systematically compiled in Table 1 for subsequent analysis.

**Table 1:** Characteristics, main findings, and methodological quality of included Mendelian randomization studies on insomnia and various diseases.

No.	Study ID (First Author, Year)	MR design	Population (ancestry)	Sample size (Exposure /Outcome)	Instrumental variables (No. of SNPs)	Exposure	Outcome	Main MR result (IVW; OR/β, 95% CI, *p*-value)	Key sensitivity analyses reported	Quality assessment (NOS-MR)
Cardiovascular diseases										
1	Yuan et al., 2021 (17)	Two-sample	European (UKB, 23andMe)	Insomnia: ~1.33 M; Outcomes: varying	142	Insomnia	Coronary Artery Disease (CAD)	OR = 1.19 (1.12–1.24), *p* = 5.4 × 10^−11^	MR-Egger, Weighted median, MR-PRESSO	High (7/8)
4	Zhang et al., 2024 (18)	Meta-review & MR	Mixed (from MR studies)	N/A (Review)	N/A (Review)	Insomnia	Composite CVD	Summary OR from meta-analysis	N/A (Review)	N/A
2	Zhang et al., 2024 (19)	Two-sample	European (UKB)	~1 M	142 (for insomnia)	Insomnia	Atrial Fibrillation (AF)	OR = 1.10 (1.02–1.15), *p* < 0.006	MR-Egger, Weighted median	Moderate (6/8)
3	Arora et al., 2023 (20)	Two-sample	European (UKB)	Sleep traits: ~452 K; MI: 43,676 cases	78–248 (for sleep traits)	Insomnia	Acute Myocardial Infarction (MI)	OR = 1.13 (1.04–1.22), *p* < 0.05	MR-Egger, Weighted median, Cochran‘s Q	Moderate (6/8)
Metabolic diseases										
5	Xiuyun et al., 2022 (27)	Two-sample, Network MR	European (UKB, 23andMe)	Insomnia: ~1.02 M; T2D: ~80 K cases	248	Insomnia	Type 2 Diabetes (T2D)	OR = 1.18 (1.11–1.25), *p* < 0.001	MR-Egger, Weighted median, MR-PRESSO, Mediation	High (7/8)
6	Liu et al., 2022 (30)	Two-sample	European (UKB)	Sleep traits: ~452 K; HbA1c: ~146 K	78 (for insomnia)	Insomnia	Glycated Hemoglobin (HbA1c)	β = 0.04% (0.02–0.06), *p* = 0.002	MR-Egger, Weighted median, Cochran‘s Q	High (7/8)
7	Liu et al., 2024 (29)	Two-sample	European (FinnGen, UKB)	Insomnia: ~1.3 M; GDM: 5,687 cases	248	Insomnia	Gestational Diabetes (GDM)	OR = 1.27 (1.13–1.43), *p* < 0.001	MR-Egger, Weighted median, MR-PRESSO	High (7/8)
8	Hayes et al., 2023 (32)	Two-sample, Bidirectional	European (UKB, GIANT)	~700 K (for adiposity traits)	248 (insomnia); ~500 (BMI)	Insomnia ↔ Adiposity	Body Mass Index (BMI)	β = 0.32 kg/m^2^ (0.21–0.43), *p* < 0.001	MR-Egger, Weighted median, Steiger filtering	Moderate (6/8)
9	Luo et al., 2024 (42)	Two-sample, Mediation	European (UKB, DIAGRAM)	SGLT2i mimicry: NR; Insomnia: ~1.3 M	Functional variants in *SLC5A2*	SGLT2 inhibition (Genetic mimicry)	Insomnia	OR = 0.78 (0.69–0.88), *p* = 0.003	MR-Egger, Weighted median, Mediation	High (7/8)
Musculoskeletal & pain disorders										
10	Ni et al., 2022 (47)	Two-sample, Multivariable	European (UKB, ARC)	Insomnia: ~1.3 M; OA: 177,517 cases	248 (for insomnia)	Insomnia	Osteoarthritis (OA)	OR = 1.12 (1.06–1.18), *p* = 2.1 × 10^−5^	MR-Egger, Weighted median, Multivariable MR	High (7/8)
11	Gao et al., 2022 (54)	Two-sample	European (UKB)	Insomnia: ~1.3 M; RA: 14,361 cases	248	Insomnia	Rheumatoid Arthritis (RA)	OR = 1.14 (1.07–1.22), *p* = 5.8 × 10^−6^	MR-Egger, Weighted median	Moderate (6/8)
13	Broberg et al., 2022 (56)	Bidirectional, Two-sample	European (UKB, 23andMe, FinnGen)	Insomnia: ~1.3 M; Pain: ~387 K	248 (insomnia); pain-specific SNPs	Insomnia ↔ Multisite Pain	Multisite Pain	OR (Insomnia→Pain) = 1.67 (1.52–1.83), *p* < 0.001	MR-Egger, Weighted median	High (7/8)
14	Yao et al., 2023 (57)	Bidirectional, Two-sample	European (UKB, FinnGen)	NR	NR	Insomnia ↔ Site-specific Pains	e.g., Headache, Back pain	Various ORs reported, *p* < 0.05	NR	Moderate (5/8)
15	Chu et al., 2022 (58)	Two-sample	European (UKB, IHGC)	NR	NR	Insomnia	Migraine	OR = 4.29, *p* < 0.001	NR	Moderate (5/8)
Mental Disorders										
16	Zhou et al., 2023 (70)	Bidirectional, Two-sample	European (UKB, PGC)	Large-scale GWAS	GWAS-significant SNPs	Insomnia ↔ Depression/Anxiety	Anxiety, Depression	Significant ORs in both directions	MR-Egger, Weighted median	High (7/8)
17	Huang et al., 2023 (71)	Two-sample	European (PGC, UKB)	Insomnia: ~1.3 M; MDD: 170,756 cases	248	Insomnia	Major Depressive Disorder (MDD)	OR = 1.22 (1.16–1.29), *p* = 1.6 × 10^−12^	MR-Egger, Weighted median, MR-PRESSO	High (7/8)
18	Gao et al., 2023 (72)	Two-sample	European (UKB, PGC)	Large-scale GWAS	248	Insomnia	Attention-Deficit/Hyperactivity Disorder (ADHD)	OR = 1.74 (1.26–2.41), *p* = 0.001	NR	Moderate (5/8)
Central nervous system diseases										
19	Guo et al., 2022 (75)	Two-sample	European (MEGASTROKE, UKB)	Insomnia: ~1.3 M; Stroke: 67,162 cases	248	Insomnia	Any Stroke	OR = 1.05 (1.01–1.09), *p* = 0.008	MR-Egger, Weighted median	Moderate (6/8)
20	Anderson et al., 2021 (77)	Bidirectional, Two-sample	European (IGAP, UKB)	NR	NR	Insomnia ↔ Alzheimer‘s Disease	Alzheimer’s Disease (AD)	OR ~1.05, *p* = 0.283 (ns)	MR-Egger, Weighted median	High (7/8)
21	Liu et al., 2023 (78)	Two-sample, Metabolomics	European (UKB, AD/PD consortia)	NR	NR	Insomnia	Alzheimer‘s & Parkinson’s Disease	Association via metabolite disruption	MR-Egger, Weighted median	High (7/8)
Cancers										
22	Liu et al., 2022 (79)	Two-sample	European (UKB, various consortia)	Insomnia: ~1.3 M; Cancers: varying	248	Insomnia	Lung Cancer, others	Significant for lung, thyroid, etc. (site-specific ORs)	MR-Egger, Weighted median, Bonferroni correction	High (7/8)
23	Shen et al., 2023 (81)	Two-sample, Mediation	European (ILCCO)	Insomnia: ~1.3 M; Lung Cancer: 29,266 cases	248	Insomnia	Lung Cancer	OR significant, *p* < 0.05	MR-Egger, Weighted median, Mediation	High (7/8)
24	Li et al., 2022 (80)	Bidirectional, Two-sample	European (UKB, FinnGen)	NR	NR	Insomnia ↔ Thyroid Cancer	Thyroid Cancer	Significant in one direction (Cancer→Insomnia)	NR	Moderate (5/8)
25	Wang et al., 2021 (82)	Two-sample	European (UKB, 23andMe)	NR	NR	Insomnia	Endometrioid Ovarian Cancer	OR = 1.60 (1.05–2.45), *p* = 0.028	NR	Moderate (5/8)
26	Du et al., 2022 (83)	Two-sample	European (UKB, FinnGen)	NR	NR	Insomnia	Bladder Cancer	No significant association (*p* > 0.05)	IVW, MR-Egger, Weighted median	High (7/8)
27	Yang et al., 2023 (89)	Two-sample, Mediation	European (UKB, FinnGen)	NR	NR	Insomnia	Primary Liver Cancer	OR significant, *p* < 0.01	MR-Egger, Weighted median, Mediation	High (7/8)
28	Hayes et al., 2023 (85)	Two-sample (Meta-MR)	European (BCAC, UKB)	NR	NR	Insomnia	Breast Cancer-Specific Mortality	HR = 0.18 (0.03–1.12), *p* = 0.68 (ns)	NR	Moderate (5/8)
Digestive system diseases										
29	Zha et al., 2023 (91)	Two-sample	European (UKB, FinnGen)	Insomnia: ~1.3 M; 12 GI endpoints	248	Insomnia	Duodenal Ulcer, Gastric Ulcer, etc.	e.g., Duodenal Ulcer: OR = 1.006 (1.002–1.010), *p* = 0.009	MR-Egger, Weighted median, MR-PRESSO	High (7/8)
30	Zha et al., 2022 (92)	Bidirectional, Mediation	European (UKB, FinnGen)	NR	NR	Insomnia ↔ Peptic Ulcer	Peptic Ulcer	OR = 1.012 (1.005–1.019), *p* = 0.002	MR-Egger, Weighted median, Mediation	High (7/8)
31	Bao et al., 2023 (93)	Two-sample, Mediation	European (MiBioGen, UKB)	Gut microbiota: 18,340; IBS: 53,400 cases	GWAS-significant SNPs for taxa	Gut Microbiota → Insomnia → IBS	Irritable Bowel Syndrome (IBS)	Significant mediation effect	MR-Egger, Weighted median, Mediation MR	High (7/8)
32	Li et al., 2022 (95)	Two-sample	European (MiBioGen, UKB)	Gut microbiota: 18,340; Insomnia: ~1.3 M	GWAS-significant SNPs for taxa	Specific Gut Bacteria (e.g., Ruminococcus)	Insomnia	e.g., Ruminococcus: OR = 0.976, *p* = 0.044	MR-Egger, Weighted median	High (7/8)
33	Wang et al., 2024 (96)	Two-sample	European (MiBioGen, UKB)	Gut microbiota: 18,340; Insomnia: ~1.3 M	GWAS-significant SNPs for taxa	Specific Gut Bacteria (e.g., Negativicutes)	Insomnia	e.g., Negativicutes: OR = 1.03, *p* < 0.001	MR-Egger, Weighted median	High (7/8)
34	Fuquan et al., 2024 (99)	Two-sample	European (OpenGWAS, FinnGen)	Gut metabolic pathways: NR; Insomnia: ~1.3 M	GWAS-significant SNPs for pathways	Gut Microbial Metabolic Pathways	Insomnia	Positive correlation reported for specific pathways	MR-Egger, Weighted median	High (7/8)
Other (telomere, pregnancy, immune)										
35	He et al., 2023 (104)	Two-sample, Sex-stratified	European (UKB)	Telomere Length: ~472 K; Insomnia: ~1.3 M	~130 (for telomere length)	Telomere Length	Insomnia	OR = 0.89 (0.84–0.95), *p* = 3.4 × 10^−4^	MR-Egger, Weighted median, Sex-specific	High (7/8)
36	Yang et al., 2022 (107)	Two-sample	European (UKB)	~87,000 mother–child pairs	NR	Insomnia	Miscarriage	OR = 1.60 (1.20–2.13), *p* = 0.001	NR	Moderate (5/8)

Quality Assessment (NOS-MR): Based on a modified Newcastle-Ottawa Scale adapted for MR studies. High (7–8): Strong instruments (F-statistic >10 typically), comprehensive sensitivity analyses (MR-Egger, weighted median, etc.), proper handling of pleiotropy and heterogeneity. Moderate (5–6): Core MR methods applied, but some limitations in sensitivity analyses, reporting, or instrument strength. Low (≤4): Major methodological concerns (e.g., weak instruments, pleiotropy not assessed).

CI, Confidence Interval; GWAS, Genome-Wide Association Study; IVW, Inverse Variance Weighted; MR, Mendelian Randomization; NOS, Newcastle-Ottawa Scale; NR, Not Reported; OR, Odds Ratio; SNP, Single Nucleotide Polymorphism; UKB, UK Biobank.

## 3 The relationship between cardiovascular diseases and insomnia

An increasing number of epidemiological and genetic studies have confirmed a clear causal association between insomnia and various cardiovascular diseases (CVDs), a relationship that may be partially mediated by factors such as body mass index (BMI) and smoking behavior (16). Mendelian randomization (MR) studies, utilizing genetic instrumental variables, provide strong evidence for the potential causal link between the two. Among these, the association between insomnia and coronary heart disease (CHD) is most conclusive. Large-scale MR analyses demonstrate that genetically predicted insomnia significantly increases CHD risk (see Table 1 for details) (17) Zhang X et al.’s (18)MR study further validated insomnia’s causal role as an independent risk factor for multiple CVDs, with its effects remaining robust despite significant confounding by age, underlying diseases, and other factors. Some researchers (19) employed MR analysis to focus on specific CVD subtypes, revealing that genetic susceptibility to insomnia is significantly associated with an increased risk of atrial fibrillation (AF) onset (*p* < 0.006). Additional observational studies further confirmed that among individuals with various sleep disorders, those with insomnia symptoms exhibited the highest risk of acute myocardial infarction (AMI) (20), reinforcing the close association between insomnia and CVDs.

The pathophysiological mechanism primarily revolves around the core concept of “hyperarousal.” Insomnia leads to sustained activation of the sympathetic nervous system and the hypothalamic–pituitary–adrenal (HPA) axis, triggering dysregulation in catecholamine and cortisol secretion. This results in accelerated heart rate, elevated blood pressure, and vascular endothelial injury, directly promoting atherosclerosis (21–25). Concurrently, insomnia-related neuroendocrine dysregulation (e.g., decreased leptin, elevated ghrelin) disrupts energy metabolism, increasing obesity risk. This indirectly damages cardiovascular health through insulin resistance and chronic inflammation, forming a vicious cycle of “insomnia-obesity-CVD” (26). Genetic studies have also revealed that certain gene loci regulating circadian rhythms and inflammation are associated with both insomnia and CVD susceptibility, suggesting a shared genetic basis (27).

Current MR evidence indicates that the causal impact of insomnia on CVD exhibits variability. Its risk effects on CHD and AF appear relatively robust. However, for specific subtypes such as AMI, MR evidence remains insufficient, and observational studies may overestimate the association. Future research should validate these relationships in larger samples and further elucidate the specific contributions of mediating factors like BMI and inflammation.

## 4 Metabolic diseases and insomnia

The bidirectional interaction between insomnia and metabolic disorders (such as type 2 diabetes, obesity, and dyslipidemia) has been validated through multidimensional epidemiological, genetic, and mechanistic studies. Among these, MR studies leverage the natural randomness of genetic markers to effectively circumvent confounding biases and reverse causality issues, further clarifying the causal direction, effect magnitude, and core mediating mechanisms between the two (see Table 1 for details).

Robust evidence indicates that genetically predicted insomnia is a causal risk factor for type 2 diabetes and worsening glycemic control (elevated HbA1c) (28, 29). The underlying mechanisms involve cortisol rhythm disruption induced by insomnia, chronic low-grade inflammation (e.g., elevated IL-6 and CRP), and the resulting decline in insulin sensitivity (30, 31). Similarly, MR studies support a unidirectional causal effect of insomnia on increased body mass index, with no established reverse pathway (32). This likely occurs through altered appetite-regulating hormones (reduced leptin, elevated ghrelin) promoting increased caloric intake, synergizing with inflammatory factors to enhance fat accumulation (33, 34). Regarding dyslipidemia, insomnia may indirectly elevate risk by activating the HPA axis, altering dietary preferences (e.g., increased high-fat food intake), and exacerbating behaviors like smoking and alcohol consumption (35–40).

The causal relationship between insomnia and T2D/obesity is strongly supported by MR evidence, though effect sizes remain generally modest (OR typically ranging from 1.1 to 1.3). The association between insomnia and dyslipidemia is largely indirect, with weaker MR evidence. Given that most current studies are based on European populations, caution is warranted when extrapolating these findings.

Based on this evidence, a “sleep-metabolism” co-management strategy can be developed: - Incorporate sleep assessment (e.g., Pittsburgh Sleep Quality Index, PSQI) into management of high-risk populations for metabolic diseases. Monitor BMI, HbA1c, and inflammatory markers (IL-6, CRP) every 6–12 months in insomnia patients (41). For T2DM patients with insomnia, prioritize SGLT2 inhibitors (e.g., dapagliflozin) due to their ability to regulate plasma Ap4A, thereby simultaneously improving metabolism and sleep (42); For obesity-related insomnia, employ cognitive behavioral therapy for insomnia (CBT-I) combined with low-dose IL-6 receptor antagonists (e.g., tocilizumab) to disrupt inflammation-mediated vicious cycles (43); For dyslipidemia-related indirect pathways, intensify low-fat dietary interventions (≤30% daily caloric intake from fat) and smoking cessation/alcohol restriction in insomnia patients to reduce overlapping common risk factors (44–46).

## 5 Musculoskeletal disorders and insomnia

MR studies suggest that insomnia may be an underlying risk factor for specific musculoskeletal disorders. Evidence indicates a causal association between genetically predicted insomnia and increased risk of osteoarthritis and low back pain (47, 48). However, no direct causal link has been established between insomnia and rheumatoid arthritis; nonetheless, insomnia may create a susceptible environment for joint inflammation by elevating systemic inflammatory markers such as C-reactive protein (CRP) (49).

Preliminary MR evidence supports an association between insomnia and musculoskeletal pain (particularly osteoarthritis), though the effect size is small. Evidence for a direct genetic causal relationship between insomnia and rheumatoid arthritis or bone mineral density is insufficient or negative.

Its potential mechanisms are closely linked to chronic inflammation. Insomnia significantly upregulates pro-inflammatory cytokines like IL-6 and TNF-*α* (50), which not only suppress osteoblast activity and affect bone density but also serve as key drivers of synovial inflammation and cartilage degradation in rheumatoid arthritis and osteoarthritis (51–54). Although current research has preliminarily revealed the association patterns and partial mechanisms between sleep and skeletal health, critical gaps remain: existing evidence predominantly focuses on macro-level correlations and classical inflammatory pathways, while the specific molecular mechanisms by which insomnia regulates bone metabolism remain unclear. Future studies could integrate single-cell RNA sequencing to analyze differentially expressed genes in osteoblasts/osteoclasts under insomnia conditions (e.g., RUNX2, RANKL); Concurrently, epigenetic studies should explore the role of DNA methylation (e.g., NR3C1 gene methylation) in insomnia’s impact on skeletal health. Deepening our understanding of core pathways through these cutting-edge technologies will provide theoretical foundations for developing targeted clinical interventions and disease prevention strategies along the sleep-bone health axis.

## 6 Pain disorders and insomnia

Pain is an unpleasant subjective emotional experience associated with tissue damage or the threat of tissue damage (55). Sleep and pain exhibit a close and complex bidirectional interaction. MRI studies confirm a significant bidirectional causal relationship between insomnia and various chronic pain conditions (e.g., widespread pain, headache, back pain), though the effects are asymmetric: insomnia’s impact on pain is stronger than pain’s reverse effect on insomnia (56, 57). Furthermore, insomnia has been identified as a distinct risk factor for migraine (58).

The neurobiological basis of this interaction involves multiple systems. Insomnia can lead to reduced endogenous opioid system function, decreased serotonin levels, and cortisol dysregulation caused by HPA axis disruption, collectively resulting in lowered pain thresholds and pain sensitization (59–61). Clinical evidence indicates that improving insomnia in patients with knee osteoarthritis is associated with concurrent enhancements in physical function, reduced knee pain, and diminished IL-6 responses to experimental pain stimuli (62), suggesting that sleep improvement may alleviate chronic pain by regulating inflammatory mediators.

Regarding therapeutic interventions, cannabinoids have been demonstrated to improve sleep through analgesic effects (63). non-benzodiazepine hypnotics (e.g., zopiclone) improve sleep without affecting pain, whereas benzodiazepines (e.g., triazolam) improve both. This difference may relate to the selective action of non-benzodiazepines on sleep-promoting neural regions (64, 65). Additionally, melatonin demonstrates clear efficacy in improving both sleep and pain (66).

In summary, the complex interactions within the sleep-pain axis offer a multi-target approach for clinical treatment. However, the specific mechanisms involving inflammatory mediators like IL-6, as well as the clinical translational value of interventions with low-level evidence, require further validation through in-depth research.

## 7 Relationship between mental disorders and insomnia

The high co-occurrence of insomnia with mental disorders such as depression and anxiety has been confirmed by extensive clinical research—the incidence of anxiety/depression symptoms among insomnia patients is significantly higher than in healthy populations, and the two conditions often mutually exacerbate each other, forming a clinical negative feedback loop of “insomnia-mental disorder” (67–69). MR studies have elucidated this complex causal network. The most compelling evidence indicates a strong bidirectional causal relationship between insomnia and anxiety disorders, where each condition exacerbates the other, creating a vicious cycle (70). Insomnia also constitutes a clear risk factor for major depressive disorder (71). Within the broader spectrum of psychiatric disorders, MR analysis indicates that insomnia increases the risk of developing attention-deficit/hyperactivity disorder and bipolar disorder, but no significant genetic causal association was found with autism spectrum disorder or schizophrenia (72). Reverse causality analysis similarly largely ruled out these psychiatric disorders as primary causes of insomnia.

The biological mechanisms underlying this comorbidity may involve shared disturbances in neuroendocrine and immune pathways, including HPA axis dysregulation, monoaminergic neurotransmitter imbalance, and neuroinflammatory responses mediated by immune mediators such as IL-6 and TNF-*α* (73). Regarding the molecular mechanisms underlying their comorbidity, studies suggest the PI3K-AKT pathway is a core inflammation-related pathway linking insomnia and depression (74). These findings provide evidence for early identification of psychiatric disorders while laying a theoretical foundation for subsequent targeted pathway drug development and clinical intervention strategies.

## 8 Relationship between central nervous system disorders and insomnia

Central nervous system disorders (stroke, Alzheimer’s disease, Parkinson’s disease, etc.) exhibit high comorbidity rates with insomnia. MRI studies suggest insomnia is a genetic risk factor for ischemic stroke, though its association with hemorrhagic stroke remains unclear (75, 76). Regarding neurodegenerative diseases, current MRI evidence does not support a direct causal relationship between insomnia and Alzheimer’s disease (77). Interestingly, daytime napping may reduce Alzheimer’s disease risk, warranting further validation. Metabolomics studies reveal shared serum metabolic dysregulation patterns between insomnia and Alzheimer’s disease/Parkinson’s disease (e.g., decreased acetylcholine, elevated propionylcholine), suggesting potential indirect co-morbidity pathways mediated by metabolism (78).

These findings not only provide evidence for risk stratification of central nervous system diseases (e.g., incorporating insomnia genetic susceptibility and serum acetylcarnitine levels into stroke/ Parkinson’s disease risk assessment), but also provide translational theoretical directions for synergistic interventions (e.g., targeting mitochondrial metabolism, optimizing daytime sleep strategies) and diagnostic biomarker screening (e.g., serum acetylcarnitine, propionylcarnitine). This holds significant clinical implications for improving the prognosis of patients with central nervous system diseases.

## 9 The relationship between cancer and insomnia

The potential association between insomnia and cancer has emerged as a research focus in epidemiology and oncology. Early observational studies, combined with MR research evidence, have identified insomnia as a potential risk factor for several cancers, including breast cancer, uterine cancer, prostate cancer, renal cancer, bladder cancer, and primary liver cancer (79, 80). The most well-established association is that insomnia increases the risk of lung cancer, with this effect partially mediated through promoting smoking and increasing body mass index (BMI) (81). Insomnia is also associated with an elevated risk of endometrioid ovarian cancer (82). However, MR analyses failed to establish a causal link between insomnia and bladder cancer, suggesting potential confounding factors in previous observational studies (83). Regarding the relationship between insomnia and thyroid cancer or breast cancer prognosis, existing MR evidence is limited or inconclusive, necessitating further research (84, 85).

Potential biological mechanisms may involve multiple pathways: insomnia disrupts circadian rhythms (e.g., by suppressing the tumor suppressor gene PER2), reduces melatonin secretion with anticancer properties, and activates pro-inflammatory pathways such as NF-κB, collectively creating a microenvironment conducive to tumor initiation and progression (86–89). Additional literature reports that melatonin can prevent tumor spread and worsened mortality by inhibiting tumor cell migration and reducing metastasis rates (90), providing a mechanistic reference for studies linking insomnia to cancer prognosis.

Integrating these MR studies on insomnia and tumors, clinical cancer treatment and management may consider measuring circadian rhythms of melatonin levels to explore dynamic sleep patterns across aging or post-cancer diagnosis. For high-risk populations like lung cancer, endometrioid epithelial ovarian cancer, or primary liver cancer patients, incorporating insomnia into risk assessment systems could prioritize sleep interventions to reduce disease incidence. For cancer patients, monitoring melatonin levels could optimize personalized treatment plans—such as exogenous melatonin supplementation to synergistically enhance antitumor efficacy and improve sleep quality—ultimately providing theoretical foundations and practical directions for precision cancer prevention and treatment.

## 10 Relationship between digestive system diseases and insomnia

The causal association between insomnia and digestive system diseases exhibits subtype specificity. Based on MR studies, only four disease categories—including duodenal ulcers and gastric ulcers—show significant positive correlations with insomnia, with varying effect sizes and mechanisms. Key findings are summarized in Table 1. In contrast, no significant associations were observed for inflammatory bowel disease or gastroesophageal reflux disease. Robust evidence indicates that insomnia is an independent causal risk factor for peptic ulcers (91, 92). Furthermore, insomnia significantly increases the risk of irritable bowel syndrome by disrupting the gut-brain axis, with gut microbiota dysbiosis playing a key mediating role (93, 94).

Dysbiosis of the gut microbiota serves as a central mediator linking insomnia to gastrointestinal disorders. Evidence indicates reduced gut microbial diversity in insomnia patients, with altered abundance of specific genera (e.g., Ruminococcus) correlated with insomnia risk (95, 96). These bacteria influence neurotransmitters (e.g., GABA) through their metabolites (e.g., short-chain fatty acids), thereby bidirectionally regulating sleep and gut function (97–99). It should be noted that current studies predominantly rely on fecal samples, and confounding factors such as diet may affect results (100–102).

These findings suggest that sleep assessments (e.g., Pittsburgh Sleep Quality Index, PSQI) should be integrated into screening protocols for high-risk populations with gastrointestinal diseases in clinical practice. Concurrently, modulating gut microbiota (e.g., through probiotic supplementation with Ruminococcus or Lactococcus species, or optimizing dietary fiber intake) may alleviate insomnia symptoms. This provides translational theoretical foundations and practical directions for the synergistic prevention and treatment of gastrointestinal diseases and insomnia.

## 11 Relationship between other diseases and insomnia

Multiple meta-regression studies have elucidated the association between insomnia and telomere length regulation, reproductive health, and immune dysfunction. Telomere length, as a marker of cellular aging, was previously hypothesized to have a causal relationship with sleep disorders (103). However, meta-regression analysis by He et al. (104) confirmed only a genetic correlation between the two, with significant gender differences: female insomnia patients exhibited more pronounced telomere shortening. Mechanistically, estrogen upregulates telomerase reverse transcriptase gene expression. Postmenopausal decline in estrogen levels may reduce this expression, accelerating telomere shortening and amplifying genetic susceptibility to insomnia (105, 106).

The causal association between insomnia in women of childbearing age and pregnancy outcomes was preliminarily confirmed by Yang et al.’s (107) two-sample Mendelian randomization study: genetic predisposition to insomnia exhibits a positive causal association with miscarriage risk. However, this study did not report causal estimates for insomnia and other perinatal outcomes. Based on existing evidence, clinicians may prioritize cognitive behavioral therapy for women with insomnia during preconception or early pregnancy, as it improves sleep efficiency (108–111) and may reduce miscarriage risk.

In the bidirectional regulation between the immune system and sleep, MRI studies have confirmed a causal relationship between plasma transforming growth factor-*β*-induced protein and preadrenal medullin with insomnia. This mechanism exacerbates insomnia by suppressing GABAergic neuronal activity and reducing slow-wave sleep duration (112).

In summary, insomnia’s cross-system impact network is jointly constituted by its genetic correlation with telomere length, causal linkages with reproductive health, and bidirectional regulation by immune cells/plasma factors. These findings suggest that clinically, monitoring relevant biomarkers can optimize insomnia risk stratification. Concurrently, behavioral interventions and targeted drug development provide evidence for the synergistic prevention and treatment of insomnia and related disorders.

## 12 Discussion

This review synthesizes 105 MR studies, demonstrating that the genetic risk of insomnia significantly increases the probability of developing over 30 chronic diseases across more than 12 major systems. Bidirectional MR analysis confirmed the primary causal direction as “insomnia → disease,” thereby clarifying over 30% of reverse causality bias observed in observational studies. Notably, causal associations between insomnia and Alzheimer’s disease, schizophrenia, autism spectrum disorder, bladder cancer, and telomere shortening were refuted by MR evidence, highlighting MR’s corrective value in etiological inference.

Integrating 105 Mendelian randomization studies reveals that genetic susceptibility to insomnia significantly elevates the probability of developing multisystem diseases across cardiovascular, metabolic, musculoskeletal, pain, psychiatric, digestive, oncological, and reproductive systems. Bidirectional MR analysis further confirms the primary causal direction as “insomnia → disease.” Notably, associations between insomnia and Alzheimer’s disease, schizophrenia, autism spectrum disorder, bladder cancer, and telomere shortening were refuted, suggesting prior findings may stem from residual confounding or reverse causation. Further multi-omics evidence converges insomnia’s cross-system pathogenic pathways onto the “hyperarousal-metabolism-inflammation” axis, forming a molecular hub for multi-disease co-occurrence.

However, the strength of existing evidence exhibits significant variation. Causal relationships between insomnia and coronary heart disease, anxiety/depression, type 2 diabetes, lung cancer, and chronic pain are supported by relatively robust MR evidence. In contrast, associations between insomnia and certain cancer subtypes, musculoskeletal disorders (excluding osteoarthritis), and gastrointestinal diseases (excluding peptic ulcers) are based on preliminary or limited evidence, requiring larger-scale studies for confirmation. Additional omics evidence converges the cross-system pathogenic pathways of insomnia onto the “hyperarousal-metabolism-inflammation” axis. Based on this, clinical consideration should be given to implementing sleep screening for high-risk populations. Interventions should center on cognitive behavioral therapy, with complementary therapies targeting specific pathways (e.g., melatonin supplementation, anti-inflammatory approaches) explored.

Evidence included in this review has limitations. First, dominant GWAS data originate from European populations, limiting the universality of conclusions. Second, insomnia definitions rely heavily on subjective reports, lacking objective measurement. Finally, current MR methods lack sufficient power for analyzing complex mediators like gut microbiota. Future efforts should establish multi-ethnic cohort studies combining objective sleep monitoring with multi-omics technologies, apply more sophisticated multivariate MR models, and validate potential intervention targets through randomized controlled trials to advance precision sleep medicine.

In summary, MR evidence redefines insomnia from a “comorbid symptom” to a “modifiable etiology.” Early identification and disruption of the “hyperarousal-inflammation” vicious cycle hold promise for reducing the overall burden of multiple chronic diseases.
